# Feasibility analysis of CT-guided thermal ablation of multiple pulmonary nodules combined with intraoperative biopsy

**DOI:** 10.3389/fradi.2022.1036026

**Published:** 2023-02-07

**Authors:** Yu-Qing Shan, Hai-Yu Wang, Xiao-Ning He, Shuang-Sheng Jiang, Hui-Hui Wang, Fan-Xia Lin

**Affiliations:** Department of Medical Imaging Centre, People’s Hospital of Rizhao, Rizhao, China

**Keywords:** lung cancer, tissue biopsy, multiple primary pulmonary nodules, microwave ablation, x-ray computed

## Abstract

**Purpose:**

To analyze the safety and feasibility of computed tomography (CT)-guided thermal ablation of multiple pulmonary nodules combined with intraoperative biopsy.

**Methods:**

The data of 431 patients with 540 lung nodules undergoing CT-guided biopsy or ablation were retrospectively analyzed. Biopsy-only group (A): 107 patients (107 lesions) received CT-guided percutaneous lung biopsy only; Ablation-only group (B): 117 cases (117 lesions) only received CT-guided thermal ablation; Single focal ablation combined with biopsy group (C): 103 patients (103 lesions) received CT-guided thermal ablation combined with intraoperative immediate biopsy; Multifocal ablation combined with biopsy group (D): 104 patients (213 lesions) received CT-guided thermal ablation combined with intraoperative biopsy. The success rate of this technique was calculated, the complications were recorded, and the positive rate of pathological diagnosis of the specimens was evaluated (the tissue specimens could be confirmed as positive by pathological diagnosis).

**Results:**

All 431 patients with pulmonary nodules successfully completed the operation, and the technical success rate was 100% (431/431). In group A, hemoptysis occurred in seven cases after operation, while no hemoptysis was observed in the other groups. Pneumothorax occurred in 8 cases in group A, 14 cases in group B, 11 cases in group C, and 13 cases in group D. Hydrothorax occurred in 4 cases in group A, 7 cases in group B, 5 cases in group C and 9 cases in group D, and there were no significant differences between the groups. The positive rate of pathological diagnosis was 84.1% (90/107) in group A, 81.5% (84/103) in group C, and 82.6% (176/213) in group D, and there was no significant difference among the groups (*P* > 0.05). A total of 15 cases in group C and 23 cases in group D underwent gene testing and analysis, and the biopsy tissue samples all met quality control standards.

**Conclusion:**

CT-guided thermal ablation of multiple pulmonary nodules combined with intraoperative biopsy does not prolong the length of hospital stay or increase the risk of postoperative complications. It can meet the requirements of clinical, pathological and genetic testing, and is safe and reliable.

## 1. Introduction

Lung cancer is the most common malignant tumor in China. In recent years, with the development of lung cancer screening programs and the increase of the number of routine physical examinations, the detection rate of multiple nodules in the same patient has gradually increased, causing psychological pressure on patients. CIRSE standard has played a positive role in promoting the development of thermal ablation therapy for lung tumors (1). Image-guided thermal ablation (IGTA) has been used to treat primary and metastatic lung tumors, and the number of patients treated is increasing rapidly every year (2). For stage I A non-small cell lung cancer (NSCLC), IGTA is feasible for patients who cannot tolerate or refuse surgery due to various factors (3). For patients with multiple pulmonary nodules, computed tomography (CT)-guided ablation combined with biopsy is a safe and effective method for the diagnosis and treatment of pulmonary nodules (4, 5). Simple needle biopsy of pulmonary nodules often damages small pulmonary vessels or bronchi, which can cause blood or air to enter the bronchi, causing serious complications such as asphyxiated hemoptysis or air embolism and even endangering the lives of patients. Thermal ablation can occlude the blood vessels in the ablation area and has a hemostatic effect, so thermal ablation combined with biopsy can reduce the risk of complications (6). However, the biopsy tissue may be completely solidified and carbonized after thermal ablation, which affects the accuracy of pathological diagnosis. Multiple ablation surgery to remove multiple pulmonary nodules can extend the length of hospital stays, reduce compliance, and increase the risk of complications. In contrast, ablation of multiple pulmonary nodules combined with immediate intraoperative biopsy could theoretically avoid these problems. This study focused on the safety and feasibility of CT-guided thermal ablation of multiple pulmonary nodules combined with intraoperative biopsy.

## 2. Materials and methods

### 2.1. Patients

The data from a total of 431 patients with pulmonary nodules who underwent CT-guided thermal puncture ablation and/or biopsy in the People's Hospital of Rizhao from April to July 2022 were retrospectively analyzed. There were 231 men and 200 women, with an average age (±Standard Deviation) of 55.4 ± 13.7 years. A total of 540 nodules were included, including 270 solid nodules and 270 ground glass nodules (GGN). All patients underwent contrast-CT within one week before operation to observe the blood supply of pulmonary nodules. The choice of treatment plan was made by MDT (thoracic surgery, oncology, respiratory, radiotherapy, imaging, pathology, etc.), and shared decision making (SDM) was performed when necessary. The patients were divided into four groups: the biopsy-only group (A) contained 107 patients (107 lesions) who received CT-guided percutaneous lung biopsy only; the ablation-only group (B) contained 117 patients (117 lesions) who only received CT-guided thermal ablation; the single-focal ablation combined with biopsy group (C) contained 103 patients (103 lesions) who received CT-guided thermal ablation combined with intraoperative immediate biopsy; and the multifocal ablation combined with biopsy group (D) contained 104 patients (213 lesions, 2 nodules in the ipsilateral lung in 99 patients and 3 nodules in the ipsilateral lung in 5 patients) who received CT-guided thermal ablation combined with intraoperative biopsy. Patients and their families were informed about the relevant matters and signed the informed consent form before the operation.

The inclusion criteria were as follows: Patients with T1N0M0 were selected according to the TNM stage of the International Association for the Study of Lung Cancer (IASLC); Maximum diameter of nodules ≤3 cm; The maximum diameter or volume of nodules increased during follow-up; The lesions were stable, but the realistic component or solid component increased; Other malignant signs are present, such as lobulation sign, burr sign, pleural depression sign, air bronchial sign, vascular cluster sign, and vascular distortion or dilation in the nodules; Patients could not tolerate or were unwilling to undergo surgery; and patients stopped taking anticoagulants at least 7 days before surgery. The exclusion criteria were as follows: patients with coagulation and cardiopulmonary dysfunction or strong desire for ablation.

### 2.2. Instruments and methods

CT was performed using a 64-detector row scanner (SOMATOM go.Up; Siemens Healthineers, Erlangen, Germany). Scanning parameters: tube voltage, 120 kV; tube current, 285 mAs; layer thickness, 5 mm; reconstruction layer thickness, 2 mm. The ECO-100A1 microwave ablation therapy instrument and matching disposable microwave ablation needle (ECO-100AL5; ECO Medical Instruments, Nanjing, China) were used. A disposable semi-automatic biopsy needle (DSN4537; Gallini S.r.l., Mantova, Italy) and a matching 17G/11G or 19G/11G coaxial positioning needle were used as the biopsy equipment.

In group A, the puncture point of the body surface was determined after chest CT scan, the needle route was designed, local disinfection was performed, and local anesthesia was performed with 10 ml 1% lidocaine. Under CT guidance, a semi-automatic biopsy needle was used to puncture into the chest and cut samples, two intact tissue samples about 10 mm long were removed and fixed in formaldehyde solution before being sent for examination. After that, chests CT scans were performed to observe lesions and any complications.

The preoperative preparation of group B was the same as above, the coaxial positioning needle reached the proximal part of the target lesion under the guidance of CT, and then the ablation needle was moved to the location of the lesion, and ablation was performed with low power (30W–45W) for a short time (2–5 min). When the density of lung tissue around the lesion increased and the halo was 5–10 mm beyond the lesion range, the ablation was stopped, the ablation needle was removed, and a chest CT scan was performed to observe whether there were complications such as pneumothorax or bleeding.

The preoperative preparation and ablation treatment of group C were the same as above, and the single-needle puncture route was designed. Firstly, CT-guided puncture biopsy needle was inserted into the lesion and cut and sampled. Two complete tissue samples about 10 mm in length were obtained. Subsequently, the coaxial needle remained in place, and the ablation needle was moved to the lesion site under the guidance of CT for pre-ablation. The ablation needle was kept in place, and then, according to the imaging findings, the ablation area was cut and sampled by puncture with a coaxial biopsy needle, and two complete tissue samples about 10 mm in length were removed. The ablation treatment was the same as above, chest CT scans were also performed to observe the lesions and ablations, and the observation indexes were the same as above. The ablation needle was removed and chest CT images were reviewed.

The preoperative preparation and ablation treatment of group D were the same as above, the puncture point on the body surface was determined using conventional chest CT scan, and the multi-needle route was designed to obtain two complete tissue samples of about 10 mm in length respectively, which were fixed in formaldehyde solution and sent for examination, chest CT scans were also performed to observe the lesions and ablations, and the observation indexes were the same as above. The ablation needle was removed and chest CT images were reviewed.

### 2.3. Pathological examination and genetic testing

Hematoxylin and eosin staining, immunohistochemical staining, and gene detection were performed on the biopsy specimens. The quality of gene test specimens was evaluated. The quality control criteria were: a total amount of DNA extracted ≥30 ng, average sequencing depth ≥1,000×, sequence return rate/coverage ≥95%, and base quality Q30 ratio ≥80%.

### 2.4. Index of observation

Taking the successful completion of the operation as a technical success, we calculated the technical success rate; the incidence of massive pneumothorax (a gas width between the pleural line and the top of the chest wall ≥3 cm or persistent pneumothorax) and immediate hemoptysis were recorded. The specimens were required to meet defined pathological diagnostic criteria, and the positive rate of pathological diagnosis and the quality of genetic test specimens were evaluated.

### 2.5. Statistical analysis

SPSS (v. 26.0; IBM Corp., Armonk, NY, United States) was used for statistical analysis. All parameter values were tested for normality and homogeneity of variance, and the data are expressed as the mean ± Standard Deviation. Analysis of variance was used for comparison among multiple groups, and the least significant difference test was used for pairwise comparison. Count data were compared using the chi-squared test. *P*-values < 0.05 were considered statistically significant.

## 3. Results

### 3.1. Basic information

There were no significant differences in sex, age, nodule nature, maximum nodule diameter, and distance between nodule and pleura among the four groups (*P* > 0.05; Table 1).

**Table 1:** Basic data of patients undergoing percutaneous thermal ablation and/or biopsy.

Group	Sex male/female	Age	Nodules nature		Maximum diameter of nodules (cm)	Minimum distance between nodule and visceral pleura
			Solid	GGN		
A. Biopsy-only	57/50	56.92 ± 8.34	56	51	1.67 ± 0.39	3.87 ± 0.66
B. Ablation-only	62/55	57.71 ± 6.81	53	64	1.64 ± 0.38	3.90 ± 0.63
C. Single-focal ablation combined with biopsy	54/49	56.37 ± 7.07	54	49	1.79 ± 0.61	3.86 ± 0.74
D. Multifocal ablation combined with biopsy	58/46	55.90 ± 8.48	107	106	1.75 ± 0.58	3.90 ± 0.69
*F*/χ^2^ value	0.276	1.137	1.515	2.056	0.544
*P-*value	0.965	0.334	0.679	0.105	0.652

GGN, ground-glass nodules.

### 3.2. Technical success rate and complications

**Figure 1:**
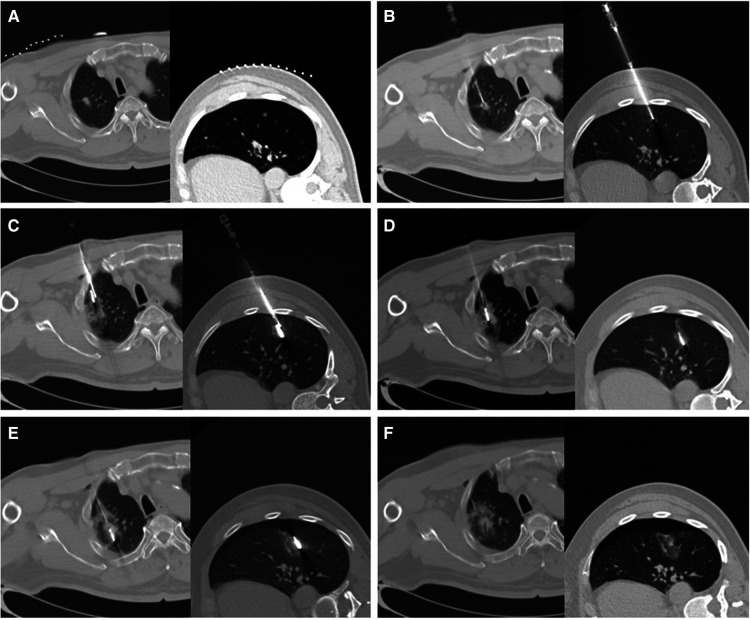
Case presentation. A 64-year-old female, presented with solid nodules in the right upper lobe and ground glass nodules in the left lower lobe. (**A**) Axial CT images of nodules in the upper lobe of the right lung and the lower lobe of the left lung. (**B**) Under the guidance of CT, the needle was inserted into the target site of the nodules, and the samples were cut and sampled. (**C**) Under the guidance of CT, the ablation needle was entered into the target site of the nodule, and the two needles were in the same field of vision. (**D**) After pre-ablation, the samples were cut again, and the CT images were reviewed. (**E**) After adjusting the ablation parameters, continue the ablation until the ablation range is satisfactory. (**F**) Immediately after the end of the ablation, CT review showed a little bleeding and pneumothorax.

The operation was successfully completed for all patients (Figure 1), and the technical success rate was 100% (540/540). In group A, hemoptysis occurred in 7 cases after operation (7/107, 6.54%), while no hemoptysis was observed in the other groups. Pneumothorax occurred in 8 cases in group A (8/107, 7.48%), 14 cases in group B (14/117,11.97%), 11 cases in group C (11/103, 10.67%), and 13 cases in group D (13/104, 12.5%), CT-guided percutaneous catheter drainage was required. Hydrothorax occurred in 4 cases in group A (4/107, 3.74%), 7 cases in group B (7/117, 5.98%), 5 cases in group C (5/103, 4.85%), and 9 cases in group D (9/104, 8.65%). There was no significant difference in the incidence of pneumothorax and pleural effusion among the four groups (*χ*^2^*^ ^*= 1.716, *P *= 0.633) (*χ^2 ^*= 2.558, *P *= 0.465). There was no significant difference in the length of hospital stay among groups (*F* = 1.067, *P* = 0.363) (Table 2).

**Table 2:** Complications within 24 h after operation in each group (%).

Group	Pneumothorax	Pleural effusion	Hemoptysis	hospital days	Pathological positive
A. Biopsy-only	8 (7.48%)	4 (3.74%)	7 (6.54%)	5.20 ± 0.88	90 (84.1%)
B. Ablation-only	14 (11.97%)	7 (5.98%)	–	4.96 ± 1.06	–
C. Single-focal ablation combined with biopsy	11 (10.67%)	5 (4.85%)	–	5.09 ± 1.20	84 (81.5%)
D. Multifocal ablation combined with biopsy	13 (12.5%)	9 (8.65%)	–	5.07 ± 1.01	176 (82.6%)
*χ*^2^ value	1.716	2.558	21.546	1.067	0.243
*P*-value	0.633	0.465	0	0.363	0.886

### 3.3. Pathological results and genetic testing

In group A, there were 58 cases of lung adenocarcinoma (nine cases of metastatic adenocarcinoma), 10 cases of squamous cell carcinoma, 8 cases of neuroendocrine carcinoma, 7 cases of poorly differentiated carcinoma (not defined) and 8 cases of benign or other lesions; 17 cases had no definite pathological diagnosis. The positive rate of pathological diagnosis was 84.1% (90/107). In group C, there were 62 cases of lung adenocarcinoma (seven cases of metastatic adenocarcinoma), 13 cases of squamous cell carcinoma, 5 cases of neuroendocrine carcinoma and 4 cases of atypical adenomatous hyperplasia, 19 cases had no definite pathological diagnosis. The positive rate of pathological diagnosis was 81.5% (84/103). In group D, there were 61 cases (125 lesions) of lung adenocarcinoma (seven cases of metastatic adenocarcinoma), 15 cases of squamous cell carcinoma (31 lesions), 6 cases of neuroendocrine carcinoma (12 lesions), and 4 cases of atypical adenomatous hyperplasia (eight lesions); 18 cases (37 lesions) had no definite pathological diagnosis. The positive rate of pathological diagnosis was 82.6% (176/213). There was no significant difference among the three groups (*χ^2 ^*= 0.243, *P *= 0.886). In group D, 23 cases underwent gene testing and analysis, and the samples all met the quality control standards (Table 3).

**Table 3:** Genetic test results of 23 cases in multiple multifocal ablation combined with biopsy group.

Number	Maximum diameter of nodules (cm)	Pathological diagnosis	Gene mutation detected before ablation	Gene mutation detected after pre-ablation
1	2.1	Adenocarcinoma	–	KRAS
2	1.9	Adenocarcinoma	EGFR	EGFR, TP53
3	1.6	Adenocarcinoma	–	EGFR, KRAS
4	2.3	Squamous carcinoma	–	TP53, FGFR1
5	1.9	Adenocarcinoma	TP53	TP53
6	1.5	Atypical hyperplasia	–	EGFR, TP53, KRAS
7	1.7	Adenocarcinoma	–	KRAS, MDM2
8	2.1	Adenocarcinoma	TP53	EGFR, TP53
9	2.3	Adenocarcinoma	–	EGFR, BRAF
10	1.8	Adenocarcinoma	–	BRAF
11	2.4	Adenocarcinoma	–	No gene mutations were detected
12	1.8	Squamous carcinoma	EGFR	EGFR
13	2.2	No definite diagnosis	–	EGFR, TP53, BRAF
14	1.5	Adenocarcinoma	No gene mutations were detected	EGFR
15	2.6	Adenocarcinoma	–	EGFR, TP53, KRAS
16	1.7	Adenocarcinoma	–	TP53
17	2.6	Atypical hyperplasia	–	No gene mutations were detected
18	2.1	Adenocarcinoma	EGFR	EGFR, TP53
19	2.5	Adenocarcinoma	–	TP53, BRAF
20	2.2	Squamous carcinoma	–	TP53, FGFR1
21	1.9	Adenocarcinoma	–	EGFR, TP53, KRAS
22	2.0	Adenocarcinoma	–	EGFR
23	1.8	Adenocarcinoma	–	BRAF

## 4. Discussion

With the continuous improvement of quality of life and the increased prevalence of physical examination, the discovery of early tumors is increasing, especially in lung cancer. Under original treatments based on surgery and supplemented by chemotherapeutic radiotherapy, patients often experience pain, complications, and serious side effects, which affect their quality of life (7). Some patients die due to severe postoperative complications, therefore treatment to improve the quality of life has received research attention (8). Percutaneous microwave ablation uses implanted microwave electrodes in target lesions under the guidance of imaging equipment to make polar molecules in the lesions move at high speed and generate heat by friction with charged particles. Within a short period of time, the temperature rises rapidly, causing irreversible necrosis of tumor cells (9), while the healthy tissues around the tumor are only minimally damaged, if at all. This method can also significantly enhance the body's anti-tumor cell immunity (10). Studies have shown that a single electrode can completely inactivate lesions with diameter less than 3 cm using microwave thermocoagulation (8). However, the incidence of postoperative pneumothorax is 10%–60% (11), especially in elderly patients with chronic obstructive pulmonary disease, due to poor tissue elasticity or structural changes of the lung (12). There is a higher risk of complications in lung nodule biopsy alone, and the incidences of pneumothorax, pulmonary hemorrhage, and hemoptysis were 35.0–51.8%, 56%–100% and 10.6%–20%, respectively (13). Low-power, fast thermal ablation can promote microthromboses in small vessels and small airway occlusion and reduce the risk of bleeding and air embolism (14). The present study showed that ablation of multiple multifocal lung nodules combined with intraoperative biopsy did not increase the risk of pneumothorax, significantly reduced the risk of bleeding compared with biopsy alone, and reduced the incidence of complications of ablation of multiple lung nodules. Genetic mutations are the main cause of tumor heterogeneity and promote tumor progression or drug resistance. In the present study, 23 patients underwent genetic testing, and related gene mutations were detected in 21 patients. In the ablation combined with biopsy group, the biopsy samples taken before ablation and after intraoperative pre-ablation met the quality control requirements of gene testing. Gene detection was performed in six samples before ablation and after pre-ablation. Among them, the results of gene detection were consistent between preoperative and intraoperative samples in five cases, and no mutations were detected in preoperative samples in one case, while mutations in KRAS and TP53 genes were detected in intraoperative samples, which may be related to the lower content of tumor cells in preoperative samples.

This study exhibits several limitations. First, there was no immediate postoperative pain evaluation, no long-term follow-up for all patients, and some patients were lost to follow-up, so it was impossible to evaluate their clinical efficacy. Secondly, ablation is only a local treatment and does not inactivate metastatic lymph nodes. Therefore, patients with a definite diagnosis of lymph node metastasis (N1, N2) should receive combined treatment with mediastinal chemoradiotherapy to improve the treatment efficacy and survival time. Finally, nodules adjacent to the pleura, pericardium, and great vessels cannot be ablated. In the future, our study group will further focus on ablation combined with seed implantation in the treatment of subpleural, peripericardial and perivascular nodules.

## 5. Conclusions

CT-guided thermal ablation of multiple pulmonary nodules combined with intraoperative biopsy is safe and feasible and meet the requirements of pathological examination and genetic testing, does not prolong the length of hospital stay or increase the risk of postoperative complications, and indirectly reduce the hospitalization costs of patients. With developmental advances and improvement of various medical instruments and accessories, minimally invasive technology is believed to be increasingly used in the comprehensive treatment of early lung cancer in the future.

## Data Availability

The original contributions presented in the study are included in the article/Supplementary Material, further inquiries can be directed to the corresponding author.
